# Improvement of free fatty acid production using a mutant acyl-CoA thioesterase I with high specific activity in *Escherichia coli*

**DOI:** 10.1186/s13068-016-0622-y

**Published:** 2016-10-06

**Authors:** Kwang Soo Shin, Sangwoo Kim, Sung Kuk Lee

**Affiliations:** 1 School of Life Sciences, Ulsan National Institute of Science and Technology (UNIST), Ulsan, 44919 Republic of Korea; 2 School of Energy and Chemical Engineering, Ulsan National Institute of Science and Technology (UNIST), Ulsan, 44919 Republic of Korea

**Keywords:** Error-prone PCR library, *Escherichia coli*, Free fatty acid, Oleochemicals, Thioesterase

## Abstract

**Background:**

Microbial production of oleochemicals has been actively studied in the last decade. Free fatty acids (FFAs) could be converted into a variety of molecules such as industrial products, consumer products, and fuels. FFAs have been produced in metabolically engineered *Escherichia coli* cells expressing a signal sequence-deficient acyl-CoA thioesterase I (‘TesA). Nonetheless, increasing the expression level of ‘TesA seems not to be an appropriate approach to scale up FFA production because a certain ratio of each component including fatty acid synthase and ‘TesA is required for optimal production of FFAs. Thus, the catalytic activity of ‘TesA should be rationally engineered instead of merely increasing the enzyme expression level to enhance the production of FFAs.

**Results:**

In this study, we constructed a sensing system with a fusion protein of tetracycline resistance protein and red fluorescent protein (RFP) under the control of a FadR-responsive promoter to select the desired mutants. Fatty acid-dependent growth and RFP expression allowed for selection of FFA-overproducing cells. A ‘TesA mutant that produces a twofold greater amount of FFAs was isolated from an error-prone PCR mutant library of *E*. *coli* ‘TesA. Its kinetic analysis revealed that substitution of Arg^64^ with Cys^64^ in the enzyme causes an approximately twofold increase in catalytic activity.

**Conclusions:**

Because the expression of ‘TesA in *E*. *coli* for the production of oleochemicals is almost an indispensable process, the proposed engineering approach has a potential to enhance the production of oleochemicals. The use of the catalytically active mutant ‘TesA^R64C^ should accelerate the manufacture of FFA-derived chemicals and fuels.

**Electronic supplementary material:**

The online version of this article (doi:10.1186/s13068-016-0622-y) contains supplementary material, which is available to authorized users.

## Background

Among many molecules produced by microbes, fatty acids have many applications to the manufacture of biofuels, cosmetics, and pharmaceutical drugs [1–4]. There are two primary pathways for production of free fatty acids (FFAs): (1) phospholipid-derived FFAs, (2) microbial FFAs. The latter has several advantages: cost-effective production from renewable biomass, less production time, feasible extraction process, and direct conversion to oleochemicals [5–10]. These benefits of microbial production of FFAs have led to several studies aimed at increasing the FFA production through metabolic engineering and synthetic biology [11–14].

FFAs have been synthesized from acyl-ACPs—intermediates of iterative cycles of fatty acid synthetic pathway—by means of thioesterases. Among them, *Escherichia coli* acyl-CoA thioesterase I (TesA) has been frequently employed because its characteristics are well understood. TesA has Ser^10^-Asp^154^-His^157^ as a catalytic triad and belongs to the family of SGNH-hydrolases [15]. The catalytic mechanism of the enzyme is as follows: the hydroxyl group of a serine residue nucleophilically attacks a thioester bond of the acyl-ACP or acyl-CoA with the assistance of the histidine residue [16]. TesA in a leaderless form (‘TesA) has been used to produce FFAs in several studies [6, 12, 17, 18].

**Fig. 1:**
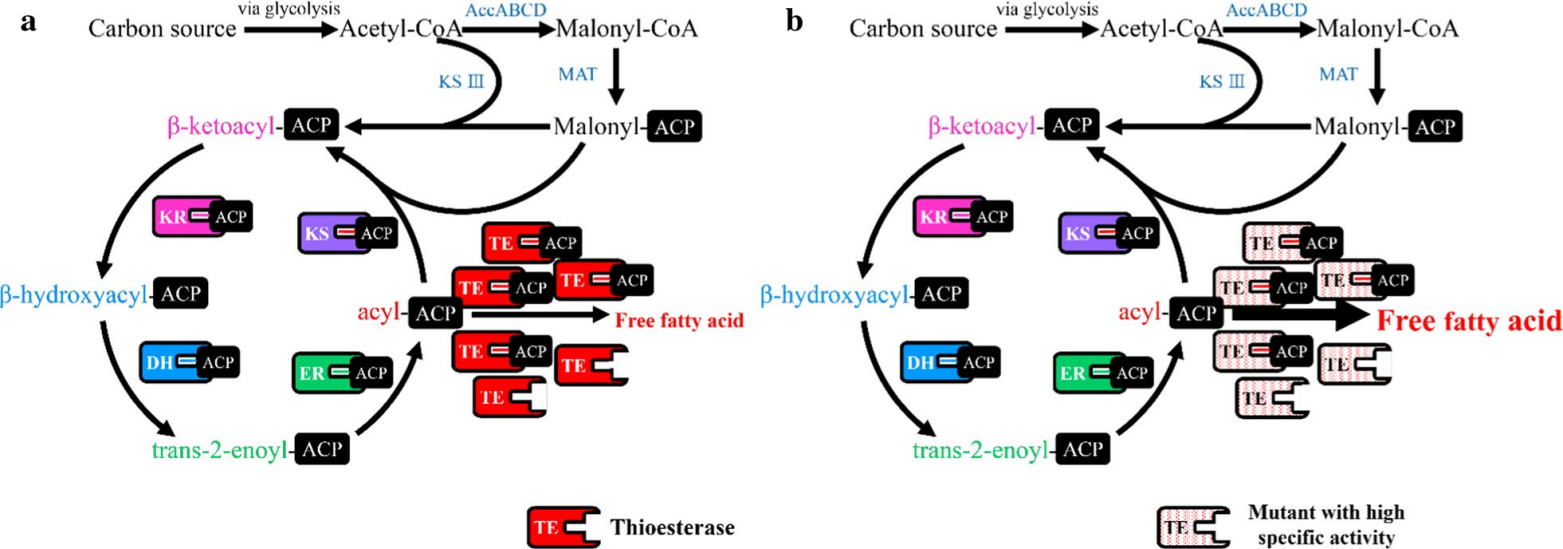
A schematic diagram of fatty acid synthesis in *E*. *coli*. **a** FFA production is improved up to a certain protein level of a wild type thioesterase; however, it does not improve further even at its highest level because of stoichiometric protein–protein interactions involved in fatty acid synthesis. **b** FFA production is improved when a catalytic active thioesterase is devised. *AccABCD* acetyl-CoA carboxylase, *MAT* malonyl-CoA:ACP transacylase, *DH* hydroxyacyl-ACP dehydratase, *ER* enoyl-ACP reductase, *KR* ketoacyl-ACP reductase, *KS III* ketoacyl-ACP synthase III, *KS* ketoacyl-ACP synthase I and II, *TE* thioesterase, *ACP* acyl carrier protein

The optimal ratio of enzymes in a metabolic pathway is believed to be important for the improvement of product titers [13, 19]. In the fatty acid synthetic pathway, nine distinct enzymes (FabA, B, D, F, G, H, I, Z, and ACP) interact with extended acyl-ACPs to synthesize long-chain acyl-ACPs. An optimal molar ratio of the fatty acid synthase (FAS) components is the key for maximization of FAS activity. It was reported that the relative molar ratio of ‘TesA to each FAS component is also required for maximal production of FFAs [18]. In addition, expression of ‘TesA enhances FAS activity by hydrolyzing the long-chain acyl-ACPs that inhibit several enzymes involved in the fatty acid synthesis [20]. According to these findings, overexpression of the wild type ‘TesA may not be sufficient to maximize the production of FFAs in *E*. *coli* (Fig. 1a). We hypothesized that engineering ‘TesA with a high specific activity is necessary for further improvement of the FFA production (Fig. 1b).

In this study, we constructed a mutant ‘TesA library through error-prone PCR and developed a high-throughput screening (HTS) method to obtain FFA-overproducing strains. The catalytic properties of the desired mutant enzyme were characterized by biochemical assays. Finally, we demonstrated that the mutant ‘TesA enzyme yielded a twofold greater amount of FFAs than the wild type enzyme did.

## Results and discussion

### Construction and characterization of a high-throughput screening system

**Fig. 2:**
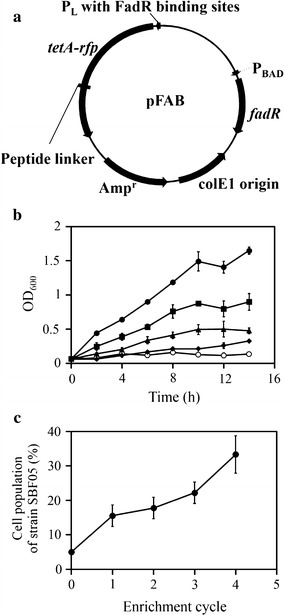
Construction of the FFA-sensing plasmid and its performance. **a** The plasmid map of the fatty acid biosensor (pFAB); *tetA*: tetracycline resistance gene; *rfp*: red fluorescent gene; Amp^r^: ampicillin resistance gene. **b** Effect of extracellular FFA concentrations on cell growth. At 40 µg/mL of tetracycline, SBF01 was cultivated with different concentrations of oleic acid (mM) [2 (*filled circle*), 1 (*filled square*), 0.5 (*filled triangle*), 0.1 (*filled diamond*), and 0 (*open circle*)]. **c** Screening efficiency. The strains SBF02 and SBF05 were cultivated in the 19:1 ratio to confirm that the FFA-overproducing cells were dominant in enrichment culture condition containing 40 µg/mL of tetracycline. The population of SBF05 was determined by colony PCR and DNA sequencing of the *fadE* locus of 15 randomly picked colonies after every enrichment cycle. *Error bars* mean standard deviations of three independent experiments

Various sensing systems were previously employed to detect specific cellular signals and to improve production of valuable chemicals [14, 21, 22]. In this study, a fatty acid-sensing system was developed based on an existing system [11] to screen FFA-overproducing mutants in the ‘TesA mutant library. We placed the TetA-RFP fusion protein rather than reporter RFP under the control of a FadR-regulated promoter, which comprised the P_L_ promoter and FadR-binding sites. This screening system was expected to allow two selections: (1) FFA-dependent tetracycline resistance for enrichment of the expected mutants and (2) FFA-dependent RFP expression to identify the desired mutants and to eliminate false positive recombinants. FadR, an acyl-CoA-responsive transcriptional regulator, represses the expression of several genes prior to binding to long-chain acyl-CoAs [23]. In *E*. *coli*, acyl-CoAs can be synthesized from FFAs by acyl-CoA ligase (FadD). Thus, the intracellular concentration of FFAs and acyl-CoAs well correlate in *E*. *coli* [11]. At the low concentrations of FFAs or acyl-CoAs, FadR may consistently inhibit the expression of TetA-RFP in our sensing system. However, the high level of FFAs or acyl-CoAs should antagonize FadR and activate the expression of TetA-RFP. This sensing system was designated as the Fatty Acid Biosensor (FAB, Fig. 2a).

First, we confirmed a dose response of the FAB toward exogenous FFAs. When *E*. *coli* MG1655 harboring pFAB, designated strain SBF01, was cultivated with various concentrations of oleic acid and 40 µg/mL of tetracycline, the fastest growth was observed in cells cultivated with the highest level of oleic acid tested (Fig. 2b). In addition, the RFP intensity of cells was well correlated with the growth of cells (Additional file 1: Figure S1), indicating that the sensing system could confer the corresponding tetracycline resistance and RFP intensity depending on the levels of FFAs. Furthermore, we measured the functionality of sensing system to internally produced FFAs. Cell growth was measured in *E*. *coli* strains MG1655 harboring pFAB (SBF01), MG1655 harboring pFAB and pBbB6c-*‘tesA* (SBF02), and MG1655 with *fadE* deletion harboring pFAB and pBbB6c-*‘tesA* (SBF05). When tetracycline (40 µg/mL) was added to exponentially growing cells, the SBF05 grew faster than other two strains (Additional file 1: Figure S2a). As expected, the SBF05 produced the highest level of FFAs, around 365 mg/L, 1.4- and 6.1-fold higher than others (Additional file 1: Figure S2b). In addition, RFP intensity was also correlated with the cell growth and FFA production of each strain (Additional file 1: Figure S2c). The fastest growth and highest RFP intensity of the SBF05 should be resulted from its high FFA production. These results imply that the constructed sensing system shows a functional response to FFAs or acyl-CoAs.

Finally, we tested whether the sensing system could act as a screening tool for the enrichment of FFA-overproducing cells. The *fadE*-undeleted strain (SBF02) and the *fadE*-deleted strain (SBF05) were mixed in the 19:1 ratio and incubated with 40 µg/mL of tetracycline. After four cycles of the enrichment, the population of the SBF05 considerably increased to 30 from 5 % after four cycles of the enrichment (Fig. 2c). This increased population of the FFA-overproducing strain (SBF05) clearly showed that the constructed sensing system detected the level of FFAs and selectively supported the growth of strains synthesizing a large amount of FFAs.

The FFA-overproducing cells showed short doubling time even at a high concentration of tetracycline (Additional file 1: Figure S2d), resulting from the increase in the ratio of the FFA-overproducing strain after four cycles of the enrichment culture (Fig. 2c). In addition, the different levels of intracellular and extracellular fatty acids resulted in changes of tetracycline resistance and RFP expression. These results indicate that the constructed sensing system effectively confers the corresponding tetracycline resistance and red fluorescence in response to FFAs produced by the cells. This genetic sensing system was used to screen ‘TesA mutants for those with high enzymatic activity that enables cells to produce more FFAs.

### Genotypic and phenotypic analysis of isolated ‘TesA mutants

**Fig. 3:**
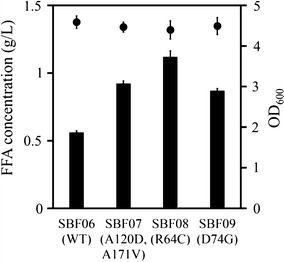
Free fatty acid production of selected strains from the ‘TesA mutant library. The expression of ‘TesA was induced with 0.3 mM IPTG, and the cells were cultivated for 48 h post-induction. Parenthesises indicate mutation of the ‘TesA. *Filled circles* indicate the optical density of each culture after 48 h. *Error bars* mean standard deviations of three independent experiments

Three recombinant cells [(MG1655 expressing a mutant ‘TesA^A120D/A171V^ (SBF07), ‘TesA^R64C^ (SBF08), or ‘TesA^D74G^ (SBF09)] produced more FFAs than MG1655 expressing a wild type ‘TesA (SBF06) (Fig. 3). The SBF07 had a mutant ‘TesA with two point mutations: A120D and A171V. It was difficult to explain how two alterations improve the FFA production because there are no studies on the Ala^171^ residue. On the other hand, the Ala^120^ residue is a part of the loop (residues 111–120) located around a substrate-binding region. The loop slightly moves when ‘TesA interacts with its substrate [24]. Thus, we can hypothesize that the mutation positively influences substrate-binding affinity and improves the FFA production. Strain SBF08, which expresses a mutant ‘TesA with a substitution of longer and basic arginine with sulfur-containing cysteine at position 64th, showed the highest production of FFAs (~1.1 g/L) among the three recombinants. This concentration was almost twofold higher than the amount produced by the SBF06. The SBF09 strain harboring ‘TesA^D74G^ produced ~1.5-fold higher FFAs than the SBF06. As an N-terminus of the flexible loop (residues 75–80) in ‘TesA, the Asp^74^ controls movements of the loop during substrate binding [24]. Thus, the alteration of Asp^74^ may increase the FFA production in a manner similar to that of the SBF07. The three mutants showed quite similar fatty acid distribution as compared with the SBF06 (Additional file 2: Table S1), indicating that the substitutions are not likely to significantly influence substrate specificity.

Alteration of enzyme characteristics such as substrate specificity [25], activity [26], and deregulated allosteric inhibition [27] has been used to increase production of desired products. The three mutants (strain SBF07, SBF08, and SBF09) isolated from the ‘TesA mutant library accounted for ~22, 33, and 22 % of 18 selected mutants, respectively. This result indicates that the constructed sensing system selectively supported the growth of FFA-overproducing cells in the mutant library. The mutations in ‘TesA^A120D/A171V^ and ‘TesA^D74G^ might cause a conformational change in the loop regions mentioned above and subsequently improve the substrate-binding affinity as previously reported [24], resulting in FFA overproduction. Given that no studies have shown the improvement of FFA production by altering amino acids in ‘TesA to date, our results may offer target amino acid residues for the engineering of ‘TesA.

### The effect of mutation at Arg^64^ of ‘TesA on enzymatic activity

**Fig. 4:**
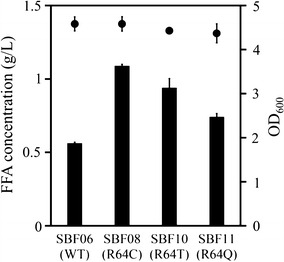
Free fatty acid production from ‘TesA mutants at position 64 generated by site-directed mutagenesis. The expression of ‘TesA was induced with 0.3 mM IPTG, and the cells were cultivated for 48 h post-induction. *Parenthesises* indicate mutation of the ‘TesA. *Filled circles* indicate the optical density of each culture after 48 h. *Error bars* mean standard deviations of three independent experiments

In order to identify the substitution leading to the highest thioesterase activity, we performed site-directed mutagenesis on the Arg^64^ residue. Replacement of arginine with threonine (‘TesA^R64T^) or glutamine (‘TesA^R64Q^) showed a 1.6- and 1.2-fold increase in the FFA production, respectively (Fig. 4). Among the cells isolated from the targeted random mutant library, a mutant with ‘TesA^R64C^-like strain SBF08 produced the largest amount of FFAs (Fig. 4). This result indicates that this substitution might provide high catalytic activity although its location is far from an active site in the protein’s structure [28]. In addition, the SBF08 showed comparable growth rate and glucose consumption rate with the SBF06 (Additional file 1: Figures S3a, b), but significantly higher FFA production (Additional file 1: Figure S3c), indicating that the expression of ‘TesA^R64C^ could improve final titer, productivity, and yield.

**Fig. 5:**
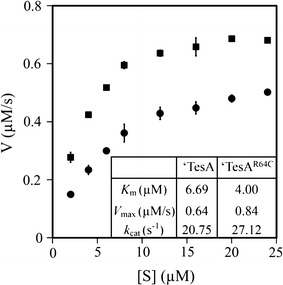
Kinetic analysis of the ‘TesA (*filled circle*) and ‘TesA^R64C^ (*filled square*). The values are average of three independent experiments. The kinetic parameters were calculated by nonlinear repression plots of the Michaelis–Menten equation. The enzyme concentration was 3.1 × 10^−5^ mM

Kinetic parameters of the ‘TesA and ‘TesA^R64C^ were analyzed in assays with palmitoyl-CoA to confirm the correlation between the increased FFA production level and increased enzymatic activity. As expected, *K*_m_ value of the ‘TesA^R64C^ toward palmitoyl-CoA was ~60 % of that of the ‘TesA, indicating that the substitution increases the substrate affinity (Fig. 5). The *k*_cat_ value of the engineered enzyme was 30 % higher than that of the wild type enzyme, suggesting that the ‘TesA^R64C^ more rapidly converts palmitoyl-CoA to palmitic acid. Therefore, these kinetic parameters can be considered supporting evidence of the highest amount of FFA synthesis in the SBF08.

Several residues of ‘TesA have been studied to understand its kinetic behavior. The movement of the switch loop (residues 75–80) influences substrate specificity by stabilizing the Michaelis complex (MC) when ‘TesA interacts with its substrate [24]. Moreover, mutation of Trp^23^ (not in the active site) significantly reduces the catalytic activity [29]. The promise of ‘TesA engineering was previously seen in the ‘TesA^L109P^, which shows altered substrate specificity [24]. Its use in engineered *E*. *coli* increased a proportion of short-chain FFAs, but not a total amount of FFAs [30]. It was previously reported that ‘TesA and its substrate rapidly form MC; however, conversion into the tetrahedral complex is slow, suggesting that ‘TesA is not likely to efficiently synthesize FFAs from acyl-ACPs [31]. Nevertheless, none of mutations in ‘TesA were found to increase the production of FFAs to date. The ‘TesA^R64C^ has higher substrate affinity and reaction rate than the wild type ‘TesA, indicating that the increased specific activity of ‘TesA improves the FFA production in *E*. *coli* as we hypothesized.

### Increased FFA production driven by high specific activity of ‘TesA^R64C^

**Fig. 6:**
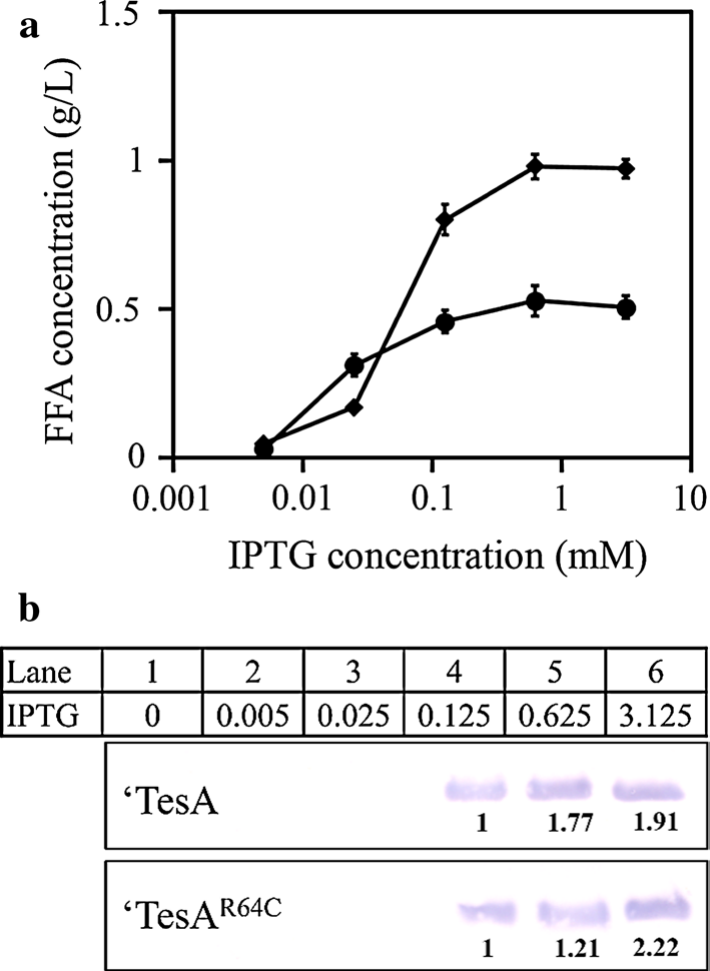
Free fatty acid production and protein expression levels at various concentrations of IPTG. **a** Free fatty acid production of strains MG1655 expressing pBbB6c-*‘tesA* ^FT^ (SBF12) or pBbB6c-*‘tesA* ^R64CFT^ (SBF13) induced with IPTG (0–3.125 mM). *Filled circles* and *filled diamonds* indicate the FFA production of the SBF12 and SBF13, respectively. *Error bars* mean standard deviations of three independent experiments. **b** Expression levels of the ‘TesA^FT^ and ‘TesA^R64CFT^ measured by western blotting. *Lane 1* without IPTG, *lane 2* 0.005, *lane 3* 0.025, *lane 4* 0.125, *lane 5* 0.625, *lane 6* 3.125 mM IPTG. The intensity values of each protein band on a western blot were determined using the ImageJ software (NIH, USA). The relative protein expression levels were calculated by dividing the intensity of each protein by the value obtained from cells induced with 0.125 mM IPTG

Multiple enzymatic activities are required to synthesize FFAs from acetyl-CoAs. It has been reported that the relative ratios of protein abundance of the FAS components are crucial for generating maximum synergy for the fatty acid synthesis [18]. Based on this fact, we hypothesized that high production of fatty acids can be achieved by optimal expression of ‘TesA with high specific activity but not by overexpression of the wild type ‘TesA. Fatty acid production was compared between cells expressing the wild type ‘TesA or the catalytically active mutant ‘TesA^R64C^ at different protein expression levels. As expected, there were slight increases in FFA production in both the strains, when they were induced with more than 0.125 mM IPTG (Fig. 6a). On the other hand, relative intensity values of each protein band were increased up to twofold in both the strains (Fig. 6b). The increase in protein levels in both the strains did not correspond to an increment in their FFA production, indicating that the thioesterase level produced even at 0.125 mM IPTG may yield the optimal ratio for the maximal FAS activity. This result also supports previous report [18] that a certain stoichiometry of FAS protein components is necessary for maximal production of fatty acids in *E*. *coli*.

In other studies, the high concentration of acyl-ACPs has been reported to cause feedback inhibition of activities of three enzymes including ACC, FabH, and FabI [32, 33]. It also alters activity of FabF and FabB: conversion of malonyl-ACP into acetyl-ACP, subsequently transformed to acetyl-CoA by an action of FabH [34]. The other substrate of ‘TesA, acyl-CoA, inhibits activity of FabI, which plays a crucial role in the fatty acid elongation [35]. These results indicate that the acyl-ACP and acyl-CoA are key regulatory signals in the fatty acid synthesis. Thus, the use of the catalytically active ‘TesA^R64C^ might accelerate the production of FFAs and their derivatives with rapid conversion of acyl-ACPs or acyl-CoAs into FFAs. This finding supported our hypothesis that the way to increase FFA production is not the overexpression of ‘TesA but the expression of more catalytically active ‘TesA.

## Conclusions

A certain ratio of ‘TesA to FAS components during FFA synthesis is necessary for efficient interaction with its substrate, acyl-ACPs, and feedback inhibition of FAS enzymes is reduced by its activity. In order to increase FFA production, the expression of a catalytically active thioesterase rather than merely excessive protein expression was tested here as a key manipulation in *E*. *coli*. Catalytically active mutant thioesterases from a ‘TesA mutant library were selected using tetracycline resistance marker for primary screening and RFP for secondary screening. The isolated ‘TesA^R64C^ mutant yielded approximately twofold higher FFA production and catalytic activity as compared with the wild type ‘TesA. The use of ‘TesA^R64C^ would boost the production of fatty acid-based chemicals and fuels because FFAs are the precious precursors for their production.

## Methods

### Bacterial strains and plasmids

Strains, plasmids, and primers used in this study are listed in Table 1 and Additional file 2: Table S2. *E*. *coli* MG1655 served as a parental strain for the construction of all the strains used in this study. Deletion of chromosomal *fadE* (acyl-CoA dehydrogenase gene) was performed by means of λ-recombination system as described elsewhere [36].

**Table 1:** Strains and plasmids used in this study

Strains and plasmids	Genotype and description	Source
Strains		
MG1655	*E*. *coli* K-12 F^−^λ^−^ *ilvG* ^−^ *rfb*-*50rph*-*1*	[40]
SBF01	MG1655 with pFAB	This study
SBF02	MG1655 containing pFAB and pBbB6c-*‘tesA*	This study
SBF03	MG1655 with *ΔfadE*::FRT	This study
SBF04	MG1655 with *ΔfadE*::FRT containing pFAB	This study
SBF05	MG1655 with *ΔfadE*::FRT containing pFAB and pBbB6c-*‘tesA*	This study
SBF06	MG1655 with pBbB6c-*‘tesA*	This study
SBF07	MG1655 with pBbB6c-*‘tesA* ^A120D, A171V^	This study
SBF08	MG1655 with pBbB6c-*‘tesA* ^R64C^	This study
SBF09	MG1655 with pBbB6c-*‘tesA* ^D74G^	This study
SBF10	MG1655 with pBbB6c-*‘tesA* ^R64T^	This study
SBF11	MG1655 with pBbB6c-*‘tesA* ^R64Q^	This study
SBF12	MG1655 with pBbB6c-*‘tesA* ^FT^	This study
SBF13	MG1655 with pBbB6c-*‘tesA* ^R64CFT^	This study
SBL01	MG1655 library containing pFAB and mutated ‘TesA	This study
BL21(DE3)	*E*. *coli* B F^−^ *ompT gal dcm lon hsdS*_*B*_(*r*_*B*_^−^ *m*_*B*_^−^) λ(DE3 *acI lacUV5*-*T7 gene 1 ind1 sam7 nin5*])	[41]
BL21(DE3)-‘TesA	BL21(DE3) with pET28a-*‘tesA*	This study
BL21(DE3)-‘TesA^R64C^	BL21(DE3) with pET28a-*‘tesA* ^R64C^	This study
Plasmids		
pBbE8a-*rfp*	ColE1 *ori*, carrying P_BAD_ promoter and *rfp*, Amp^R^	[42]
pBbE8a-*fadR*	pBbE8a with *Δrfp*::*fadR* (from MG1655), Amp^R^	This study
pBBR1 MCS-3	Cloning vactor carrying MCS and *lacZ alpha*, Tc^R^	[43]
pFAB	pBbE8a-*fadR* carrying P_LR_-*tetA*-*rfp* on *Aat*II site	This study
pBbB6c-*gfp*	BBR1 *ori*, carrying P_L-lacO1_ promoter and *gfp*, Cm^R^	[42]
pBbB6c-*‘tesA*	pBbB6c-*gfp with Δgfp*::*‘tesA*, Cm^R^	This study
pBbB6c-*‘tesA* ^A120D, A171V^	pBbB6c-*gfp with Δgfp*::*‘tesA* ^A120D, A171V^, Cm^R^	This study
pBbB6c-*‘tesA* ^R64C^	pBbB6c-*gfp with Δgfp*::*‘tesA* ^R64C^, Cm^R^	This study
pBbB6c-*‘tesA* ^D74G^	pBbB6c-*gfp with Δgfp*::*‘tesA* ^D74G^, Cm^R^	This study
pBbB6c-*‘tesA* ^R64T^	pBbB6c-*gfp with Δgfp*::*‘tesA* ^R64T^, Cm^R^	This study
pBbB6c-*‘tesA* ^R64Q^	pBbB6c-*gfp with Δgfp*::*‘tesA* ^R64Q^, Cm^R^	This study
pBbB6c-*‘tesA* ^FT^	pBbB6c- *gfp with Δgfp*::flag tagged*‘tesA,* Cm^R^	This study
pBbB6c-*‘tesA* ^R64CFT^	pBbB6c- *gfp with Δgfp*::flag tagged*‘tesA* ^*R64C*^, Cm^R^	This study
pET28a (+)	Expression vector with (His)_6_-tag, Km^R^	Novagen
pET28a-*‘tesA*	pET28a with C-terminally (His)_6_-tagged *‘tesA*	This study
pET28a-*‘tesA* ^R64C^	pET28a with C-terminally (His)_6_-tagged *‘tesA* ^R64C^	This study

To generate all the plasmids harboring *‘tesA* variants, the BioBrick plasmid pBbB6c-*gfp* served as a starting vector, and we replaced the *gfp* insert with PCR-amplified *‘tesA* alleles after digestion of the plasmid and PCR products with *Eco*RI and *Xho*I. The *‘tesA* and *tesA* ^R64C^ alleles were also cloned into the *Nco*I- and *Xho*I-digested pET28a expression vector for purification of His-tagged proteins. The biosensor plasmid pFAB was constructed by cloning *E*. *coli fadR* and a *tetA*-*rfp* fusion gene with a FadR-responsive promoter into pBbE8a-*rfp*. In brief, *fadR* of *E*. *coli* MG1655 was amplified by means of primers, FadR FP and FadR RP, and ligated into pBbE8a-*rfp* after digestion with *Bgl*II and *Xho*I, resulting in the pBbE8a-*fadR* vector. The translational fusion of *tetA* and *rfp* linked by a flexible peptide linker with GGGS × 4 [37] was ligated with P_L_ promoter and FadR-binding sites [11] using splice overlap extension (SOE)-PCR [38], and the product was cloned into the *Aat*II-digested pBbE8a-*fadR* vector.

### Media and cultivation conditions

The response of the fatty acid-sensing system to fatty acids was measured as previously described [23]. An overnight culture grown in rich broth (RB) (10 g of tryptone, 5 g of NaCl and 1 g of yeast extract per liter) was inoculated at 1:100 into 5 mL of the fresh RB medium supplemented with the various concentrations of oleic acid (0, 0.1, 0.5, 1, or 2 mM) and 40 µg/mL of tetracycline. Tween 20 was added to final concentration of 0.5 % to solubilize the oleic acid. After 24 h of growth at 37 °C, RFP fluorescence and optical density at 600 nm (OD_600_) were measured on a 96-well microplate reader (Infinite M200 TECAN, Austria) and a spectrophotometer (Libra S22, England). The fluorescence was then normalized to OD_600_ (RFP fluorescence per OD_600_ unit). The response of sensing system to internally produced FFAs was measured as cultivating various strains in the M9 medium supplemented with 2 mM MgSO_4_, 0.1 mM of CaCl_2_, additional 100 mM of sodium phosphate buffer (pH 7.0), and 0.6 % of glycerol. Addition of 0.3 mM IPTG and 40 µg/mL of tetracycline was conducted at OD_600_ of 0.5. Each parameter such as OD_600_, RFP intensity, and FFA concentration was measured as mentioned above and ‘Analysis of FFA and glucose’ section, respectively.

To analyze the FFA production, a single colony was picked from an agar plate and incubated with 5 mL of a LB medium at 37 °C. An overnight culture was washed with distilled water twice and inoculated at the inoculum size of 3 % into 5 mL of the M9 medium supplemented with 2 mM MgSO_4_, 0.1 mM CaCl_2_, additional 100 mM sodium phosphate buffer (pH 7.0), and 2 % of glucose. The cells were cultivated at 30 °C for 48 h. The expression of thioesterases was induced by the addition of 0.3 mM IPTG at OD_600_ of 0.5. To measure several parameters such as cell density, glucose concentration, and FFA concentration in the SBF06 and SBF08, cells were cultivated in mini-bioreactors as previously described [10]. Briefly, cells were grown in LB medium, which was used to inoculate 1:100 into 5 mL of the M9 medium supplemented with 2 mM of MgSO_4_, 0.1 mM of CaCl_2_, 3 % of glucose, 1 g/L yeast extract, and trace elements. The trace elements consist of 2.4 g of FeCl_3_·6H_2_O, 0.3 g of CoCl_2_·6H_2_O, 0.15 g of CuCl_2_·2H_2_O, 0.3 g of ZnCl_2_, 0.3 g of Na_2_MO_4_·2H_2_O, 0.075 g of H_3_BO_3_, and 0.495 g of MnCl_2_·4H_2_O per liter. Overnight grown cells in the minimal medium were harvested and resuspended in 70 mL of the fresh M9 medium. The pH was maintained at 7.0 with 2 N NaOH. IPTG (0.3 mM) was added to induce the expression of thioesterase at OD_600_ of 1.

### Mutant library construction

Error-prone PCR mutagenesis of *‘tesA* was conducted, and the PCR products were cloned into *Eco*RI and *Xh*oI sites of pBbB6c-*gfp* by the Mutagenex Inc (http://www.mutagenex.com). The mutant library containing 10^6^ independent colonies was constructed without hot spots of mutations.

Site-directed mutagenesis was performed by PCR amplification using the overlapping oligonucleotide primers containing degenerate codon NNS [39] to randomly replace the arginine at position 64 of ‘TesA with another amino acid. Briefly, two overlapping PCR products were generated from *tesA* by means of the primer sets (TesA FP, TesA SD RP, TesA SD FP, and TesA RP). Overlapping PCR products were then spliced using the appropriate primer set (TesA FP and TesA RP). The final constructs were obtained by the same procedure that was used for plasmid construction. In total, 1016 colonies were generated.

### Enrichment and isolation of ‘TesA mutants with increased activity

To confirm the enrichment efficiency, two strains in a 19:1 ratio (SBF02–SBF05) were mixed and incubated in the LB medium supplemented with 0.5 % of glucose, 0.1 mM of IPTG, and 40 µg/mL of tetracycline. The cells were subcultured at 1:50 into the fresh medium every 24 h. After each round of enrichment, cells from each mixed culture were plated on an agar plate and incubated at 37 °C overnight. Fifteen of the resulting colonies were randomly picked and subjected to colony PCR and DNA sequencing of the amplified *fadE* locus using primers (*fadE* seq FP and *fadE* seq RP) to evaluate the proportion of SBF05 in each mixed population. For enrichment of FFA-overproducing cells, the ‘TesA mutant library was transformed into strain SBF01, and 7.2 × 10^9^ cells harboring mutant ‘TesA were obtained. Three successive subcultures were performed by transferring 2 % (v/v) of the culture to the new subculture at 24-h intervals. The concentration of tetracycline was increased at every transfer (40, 50, and 60 µg/mL).

Cells enriched with tetracycline were sorted based on the RFP signal using a FACS Calibur instrument (BD Bioscience, USA). The sorted cells were recovered in 50 mL of the SOC medium at 37 °C for 1 h prior to plating on an agar plate. For single-cell culture analysis, colonies grown on the agar plate were cultivated in a deep 96-well plate with 200 µL of the LB medium at 30 °C for 16 h. Overnight cultures were transferred at 1:100 into a new deep 96-well plate filled with 400 µL of the LB medium supplemented with 0.5 % of glucose and 0.1 mM IPTG. After 24 h of cultivation, the RFP signal was measured as described above.

### Analysis of FFA and glucose

The concentration of FFAs was measured as previously described [6]. In a 2.0-mL tube, 500 µL of cultures was taken and acidified by the addition of 50 µL of 6 N HCl. The FFAs were extracted by vigorous vortexing for 30 s with 500 µL of ethyl acetate and 50 µL of 1 g/L methyl nonadecanoate (Sigma-Aldrich) as an internal standard. The organic layer was separated after centrifugation for 2 min, and the same procedure was repeated with additional 500 µL of ethyl acetate. Extracted FFAs were subjected to methylation with 100 µL of the mixture MeOH: 6 N HCl (9:1, v/v) and 100 µL of TMS-diazomethane (Sigma-Aldrich). After incubation for 10–15 min, fatty acid methyl esters (FAMEs) were analyzed using a gas chromatograph (Agilent 7890A) equipped with a flame ionization detector (FID) and a DB-5 column (30 m × 0.25 mm, Agilent Technologies). The identification and concentration of FAMEs were analyzed by comparing peaks with the external standards composed of C10–22 FAMEs (Sigma-Aldrich). Oven temperature of 60 °C was held for 2.5 min and ramped to 250 °C at 20 °C/min, and then held for 4 min. Finally, temperature reached 325 °C at 10 °C/min and was held for 5 min.

Residual glucose was analyzed using a Shimadzu HPLC station equipped with a refractive index detector (Shimadzu) and a SIL-20A auto-sampler (Shimadzu) as previously described [10]. Supernatant of each culture was collected and heated at 80 °C for 1 h prior to second centrifugation for 30 min. The final samples were injected into a HPX-87P column (Bio-Rad) at 0.6 mL/min with HPLC-grade water.

### Enzyme expression and kinetic analysis

BL21(DE3) cells harboring pET28a-*‘tesA* and pET28a-*‘tesA* ^R64C^ were cultured in the LB medium at 37 °C until OD_600_ reached 0.6. The expressions of ‘TesA and ‘TesA^R64C^ were induced by the addition of 0.1 mM IPTG, and the cells were allowed to grow at 18 °C for 20 h. The cells were harvested by centrifugation at 4000×*g* for 20 min, resuspended in lysis buffer (40 mM Tris–HCl, pH 8.0), and disrupted by ultrasonication (Sonic Dismembrator Model 500, Fisher Scientific). The cell debris was removed by centrifugation at 13,000×*g* for 40 min, and the supernatant was loaded onto Ni–NTA agarose (QIAGEN). The bound proteins were eluted with 300 mM imidazole in lysis buffer. The purified protein was concentrated to 3.1 × 10^−4^ mM in 40 mM Tris–HCl pH 8.0.

Thioesterase activity was measured on a UV-1800 UV–Vis Spectrophotometer (Shimadzu, Japan). The reaction mixture contained 50 mM of potassium phosphate buffer (pH 7.0), 80 µg/mL of BSA, 0.1 mM of DTNB [5,5′-dithiobis-(2-nitrobenzoic acid)], 3.1 × 10^−5^ mM of enzyme, and a substrate (0, 2, 4, 6, 8, 12, 16, 20, or 24 µM palmitoyl-CoA) in the volume of 1 mL [31]. The reduction of DTNB by released CoA was monitored at 412 nm for 1–3 min. The kinetic parameters were calculated by nonlinear repression plots of the Michaelis–Menten equation.

## Additional files

10.1186/s13068-016-0622-y Additional figures.

10.1186/s13068-016-0622-y Additional tables.

## Abbreviations

ACC: acetyl-CoA carboxylase in *E*. *coli*
ACP: acyl carrier protein
acyl-CoA: acyl coenzyme A
FabB: β-ketoacyl-ACP synthase I in *E*. *coli*
FabF: β-ketoacyl-ACP synthase II in *E*. *coli*
FabH: β-ketoacyl-ACP synthase III in *E*. *coli*
FabI: enoyl-ACP reductase in *E*. *coli*
FACS: fluorescence-activated cell sorting
FadD: acyl-CoA ligase in *E*. *coli*
FadR: multifunctional dual regulator of fatty acid metabolism in *E*. *coli*
FFA: free fatty acid
IPTG: isopropyl 1-thio-β-d-galactopyranoside
MC: Michaelis complex
OD_600_: optical density at wavelength of 600 nm
RFP: red fluorescent protein
‘TesA: leader sequence deleted thioesterase I from *E*. *coli*
TetA: tetracycline resistance protein
