# Supplementary material for: Improvement of free fatty acid production using a mutant acyl-CoA thioesterase I with high specific activity in Escherichia coli
Source: Biotechnol Biofuels. 2016 Oct 6;9:208. doi: 10.1186/s13068-016-0622-y (PMC5053343; doi:10.1186/s13068-016-0622-y)
Supplement: Supplementary file 1 — 10.1186/s13068-016-0622-y Additional figures. [file 13068_2016_622_MOESM1_ESM.docx]

**Figure S1**


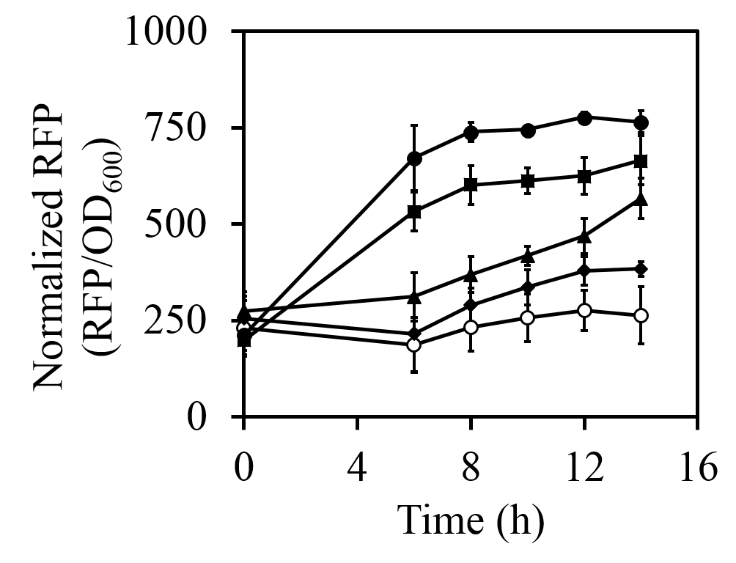


**Figure S1.** Effect of extracellular FFA concentrations on RFP expression. At 40 µg/mL tetracycline, SBF01 was cultivated with various concentrations of oleic acid (mM) [2 (●), 1 (■), 0.5 (▲), 0.1 (◆), and 0 (**○**)].

**Figure S2**


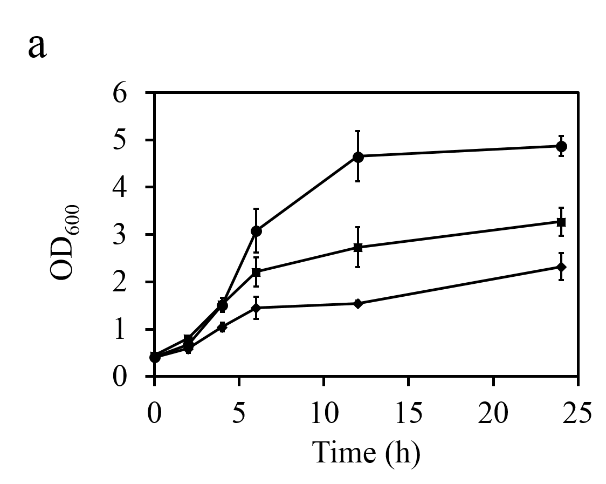

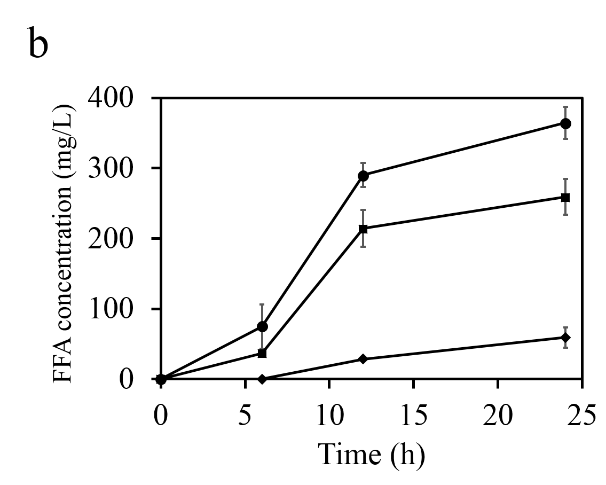


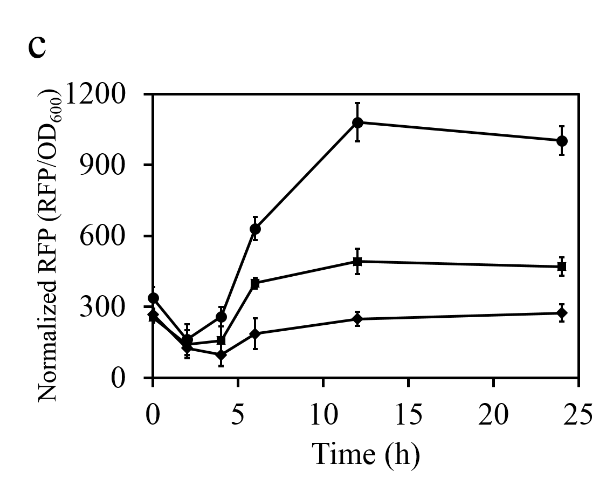

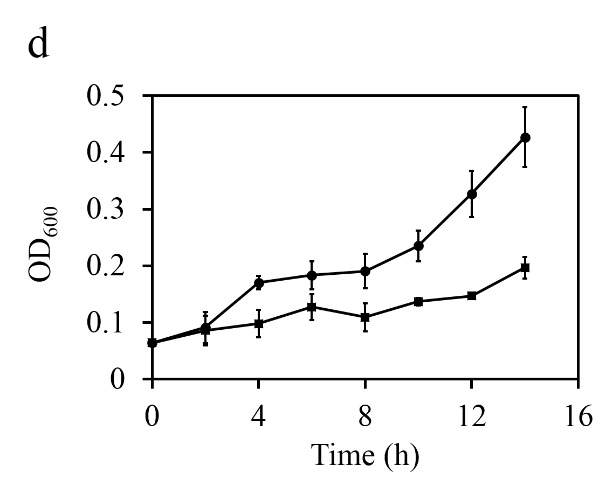


**Figure S2.** Response of the FAB biosensor to endogenous FFAs. Three strains (SBF01, SBF02, and SBF05) containing the biosensor were cultivated in minimal medium to confirm its response to internally produced FFAs. Tetracycline and IPTG were added in exponentially growing cells (around OD_600_ of 0.5, time point 0 h). (a) Cell growth of three strains. (b) Concentration of internally produced FFAs. (c) Normalized RFP intensity in three strains. (d) Effect of internally produced FFAs on cell growth in enrichment medium. The cell growth of two strains (SBF02 and SBF05) was measured to confirm enrichment. Each symbol represents SBF01 (◆), SBF02 (■), and SBF05 (●). Error bars mean standard deviations of three independent experiments.

**Figure S3**

**
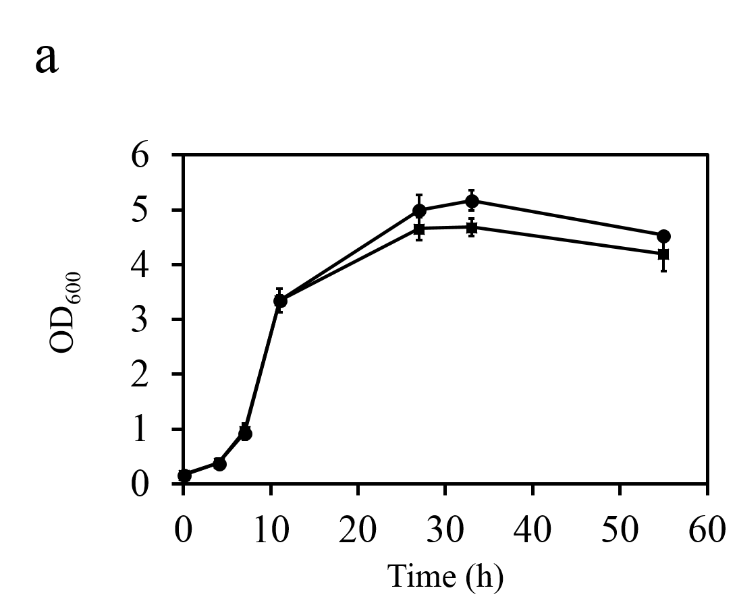

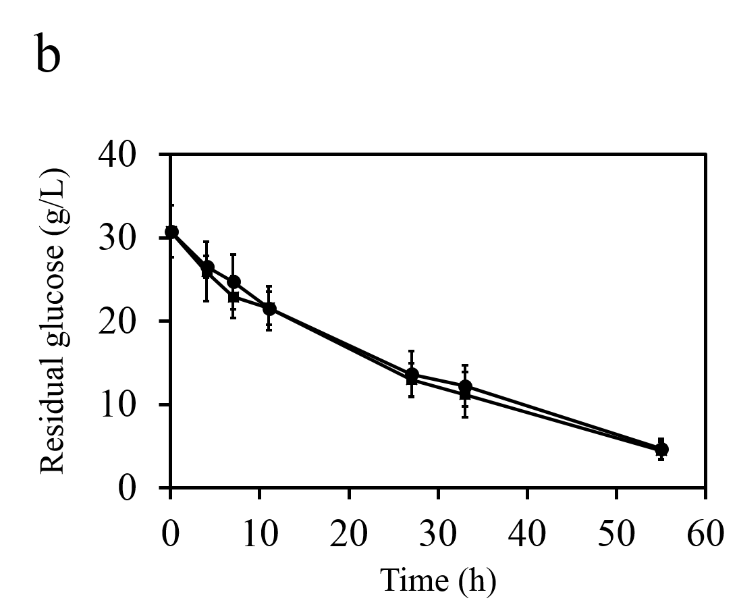

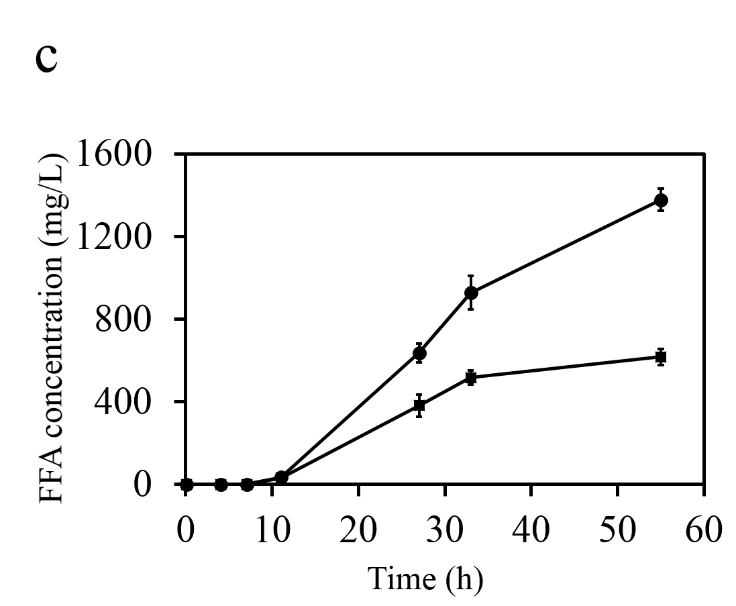
**

**Figure S3.** Batch culture of the SBF06 and SBF08 in mini-bioreactor. Two strains were cultivated in the defined M9 minimal medium (see Methods) to measure (a) cell density, (b) glucose consumption, and (c) FFA production. Each symbol represents SBF06 (■) and SBF08 (●). Error bars mean standard deviations of three independent experiments.
