# Supplementary material for: Improvement of free fatty acid production using a mutant acyl-CoA thioesterase I with high specific activity in Escherichia coli
Source: Biotechnol Biofuels. 2016 Oct 6;9:208. doi: 10.1186/s13068-016-0622-y (PMC5053343; doi:10.1186/s13068-016-0622-y)
Supplement: Supplementary file 2 — 10.1186/s13068-016-0622-y Additional tables. [file 13068_2016_622_MOESM2_ESM.docx]

**Table S1** Summary of mutant characteristics and experimental results.

| Strain | | SBF06 | SBF07 | SBF08 | SBF09 | SBF10 | SBF11 |
| --- | --- | --- | --- | --- | --- | --- | --- |
| Mutation position | | Wild type | A120D, A171V | R64C | D74G | R64T | R64Q |
| FFA (g/L) | | 0.57 | 0.92 | 1.12 | 0.87 | 0.94 | 0.74 |
| FFA distribution (%) | C12 | 22.63 | 21.14 | 19.92 | 22.04 | 25.05 | 28.79 |
|  | C14 | 42.10 | 54.59 | 56.02 | 56.46 | 59.74 | 56.58 |
|  | C16 | 27.88 | 19.32 | 19.56 | 16.65 | 10.74 | 10.46 |
|  | C18:1 | 7.39 | 4.95 | 4.50 | 4.84 | 4.47 | 4.17 |
| Final OD_600_ | | 4.56 | 4.46 | 4.39 | 4.49 | 4.43 | 4.37 |
| Relative  RFP intensity^a^ | | 1.00 | 1.21 | 1.34 | 1.17 | 1.22 | 1.11 |

^a^ Relative RFP intensity was calculated by dividing RFP intensity of the mutants by that of the SBF06.

**Table S2** Primers used in this study

| Primers | Sequence (5’-3’) |
| --- | --- |
| *fadE*_del_FP | CCATATCATCACAAGTGGTCAGACCTCCTACAAGTAAGGGGCTTTTCGTTGTGTAGGCTGGAGCTGCTTC |
| *fadE*_del_RP | TTACGCGGCTTCAACTTTCCGCACTTTCTCCGGCAACTTTACCGGCTTCGATTCCGGGGATCCGTCGACC |
| *fadE*_seq_FP | AAAAGTTAGCCAGCGTTTCCGCCGC |
| *fadE*_seq_RP | ACGTTGGGAGATGAGACGTATCAGG |
| FadR FP | AAAGATCTTTTAAGAAGGAGATATACATATGGTCATTAAGGCGCAAAG |
| FadR RP | TACTCGAGTTATCGCCCCTGAATGGCTA |
| TesA FP | AAAGAATTCAAAAGATCTTTTAAGAAGGAGATATACATATGGCGGACACGTTATTGAT |
| TesA RP | TTACTCGAGTTATGAGTCATGATTTACTA |
| TesA seq FP | AATTGTGAGCGGATAACAATTGAC |
| P_LR_ FP | ACCTGACGTCGCTAGCATCTGGTACGACCAGATTTGACAATCTGGTACGACCAGATGATACTGAGCACATCAGCAGGACGCACTGA |
| TetA FP | ACATCAGCAGGACGCACTGACCGAATTCAATTTAAGAAGGAGATATACATATGAAATCTAACAATGCGCTCATCG |
| TetA RP | AGAACCGCCTCCAGAACCACCACCGGAGCCGCCGCCGCTTCCACCGCCGGTCGAGGTGGCCCGGCTCCATGCA |
| RFP FP | GGCGGTGGAAGCGGCGGCGGCTCCGGTGGTGGTTCTGGAGGCGGTTCTATGGCGAGTAGCGAAGACGTTATCA |
| RFP RP | ATAAGACGTCTACCGCCTTTGAGTGAGCTG |
| TES_his_ FP | ATATACCATGGCGGACACGTTATTGATTCTGGGTG |
| TES_his_ RP | TGGTGCTCGAGTGAGTCATGATTTACTAAAGGCTGC |
| TES_his_ seq RP | CTAGTTATTGCTCAGCGG |
| TES_flag_ RP | CCTTACTCGAGTTACTTGTCATCGTCATCCTTGTAATCTGAGTCATGATTTACTAAAGGCTGC |
| TesA SD FP | TGCTGAAACAGCATCAGCCGNNSTGGGTGCTGGTTGAACTGGGCGGCAAT |
| TesA SD RP | CGGCTGATGCTGTTTCAGCA |

Underlined sequences indicate restriction enzyme sites.
